# Emergency Department Referral for Hospice and Palliative Care Differs among Patients with Different End-of-Life Trajectories: A Retrospective Cohort Study

**DOI:** 10.3390/ijerph18126286

**Published:** 2021-06-10

**Authors:** Victor Wei-Che Shen, Che Yang, Li-Ling Lai, Ying-Ju Chen, Hsien-Hao Huang, Shih-Hung Tsai, Teh-Fu Hsu, David Hung-Tsang Yen

**Affiliations:** 1 School of Medicine, National Yang Ming Chiao Tung University, Taipei 112, Taiwan; victorshen90066@gmail.com (V.W.-C.S.); yjchen0304@gmail.com (Y.-J.C.); hhhuang@vghtpe.gov.tw (H.-H.H.); tfhsu@vghtpe.gov.tw (T.-F.H.); 2 Department of Nursing, Taipei Veterans General Hospital, Taipei 112, Taiwan; cyang@vghtpe.gov.tw (C.Y.); lllai@vghtpe.gov.tw (L.-L.L.); 3 Department of Emergency Department, Taipei Veterans General Hospital, Taipei 112, Taiwan; 4 Institute of Emergency and Critical Care Medicine, College of Medicine, National Yang Ming Chiao Tung University, Taipei 112, Taiwan; 5 Department of Emergency Medicine, National Defense Medical Center, Taipei 114, Taiwan; tsaishihung@yahoo.com.tw; 6 Department of Nursing, Yuanpei University of Medical Technology, Hsinchu 300, Taiwan

**Keywords:** palliative care, illness trajectory, emergency department

## Abstract

Emergency units have been gradually recognized as important settings for palliative care initiation, but require precise palliative care assessments. Patients with different illness trajectories are found to differ in palliative care referrals outside emergency unit settings. Understanding how illness trajectories associate with patient traits in the emergency department may aid assessment of palliative care needs. This study aims to investigate the timing and acceptance of palliative referral in the emergency department among patients with different end-of-life trajectories. Participants were classified into three end-of-life trajectories (terminal, frailty, organ failure). Timing of referral was determined by the interval between the date of referral and the date of death, and acceptance of palliative care was recorded among participants eligible for palliative care. Terminal patients had the highest acceptance of palliative care (61.4%), followed by those with organ failure (53.4%) and patients with frailty (50.1%) (*p* = 0.003). Terminal patients were more susceptible to late and very late referrals (47.4% and 27.1%, respectively) than those with frailty (34.0%, 21.2%) and with organ failure (30.1%, 18.8%) (*p* < 0.001, *p* = 0.022). In summary, patients with different end-of-life trajectories display different palliative care referral and acceptance patterns. Acknowledgement of these characteristics may improve palliative care practice in the emergency department.

## 1. Introduction

Palliative care for people with a life-threatening illness has been widely recognized as a human right and is strongly advocated globally [1]. A large body of evidence suggests that early identification of the need for palliative care has diverse benefits, such as reduced unscheduled hospitalization [2], fewer avoidable/inappropriate admissions to the emergency department (ED) or intensive care unit [3,4], better quality of life and death [5,6], and lower stress burden and better bereavement adjustment for caregivers [7,8,9,10]. Previous studies revealed that patients with serious or life-threatening illness were likely to find themselves in an emergency department in their last month of life, many of whom were admitted and subsequently died in the hospital [11,12]. Along with the global development, Taiwan has been proactively promoting palliative care through legislation and various measures. Taiwan’s model of palliative care response in the ED was established in 2002 [13]. All types of palliative care were included in the National Health Insurance payment scheme in 2000 for cancer patients and 2009 for noncancer patients. The country’s palliative care system has grown substantially throughout the past two decades; however, little is known about the perception and acceptance of palliative care referral in the ED among patients with different end-of-life trajectories.

Although not initially considered the best setting for palliative care referral, the ED may identify early signs of palliative care needs upon early emergency visits, and on the other hand, may also be the last opportunity for patients to initiate palliative care. Thus, the realization of the importance of palliative care referral in the ED has gradually come to light [14]. Due to the unique environment of the ED, quick and precise assessments of patients are highly demanded. Studying the characteristics of patients by end-of-life trajectories may aid the understanding of their palliative care needs. Palliative care referral differs greatly by disease trajectory [15,16]. The end-of-life process can be categorized into three trajectories: terminal illness, organ failure, and frailty. Each trajectory has its own unique characteristics, including patient characteristics, patterns of functional decline, and medical resource use. According to the European Association for Palliative Care, noncancer patients may have the same palliative care needs as cancer patients, but less access and usage (40% compared with 60% for cancer patients) [17]. Not being aware of the different characteristics of end-of-life trajectories and undue optimism regarding the chances of survival may contribute to late referral for palliative care [15,18]. Hence, understanding the characteristics of different end-of-life trajectories and employing prognostic indicators can facilitate recognition of clinical situations and improve identification of patients requiring palliative care referral [19]. However, there is a dearth of studies on the differences in palliative care initiation and referral among the three end-of-stage trajectories in the ED.

Analyzing the characteristics of palliative care referral in patients with different end-of-life trajectories provides us with a better understanding of the palliative infrastructure in the ED, which may further help us establish a more reliable guideline. This study explores the timing and acceptance of palliative care referral in the ED for patients with progressive chronic conditions according to different end-of-life trajectories.

## 2. Materials and Methods

### 2.1. Study Design and Participants

This was a retrospective study conducted at a 3000-bed medical center in Northern Taiwan. Participants were aged 20 years or older and admitted to the emergency intensive care unit (EICU) within the time period of 1 February 2018 to 31 January 2020. The EICU is a subunit in the ED, which serves as a temporary intensive care unit for patients who have received emergent life-support treatment in the ED and await hospital admission for further intensive or floor care. Patients that suffer from life-threatening illnesses and require more detailed medical attention are all eligible for admission to the EICU. All patients admitted to the EICU, except those with sudden death, were subsequently screened for palliative care needs. Patients with sudden death (e.g., accident victims) were excluded from our analysis because of the diminished potential role of palliative care in their conditions.

### 2.2. Measurement

#### 2.2.1. End-of-Life Trajectory

**Figure 1:**
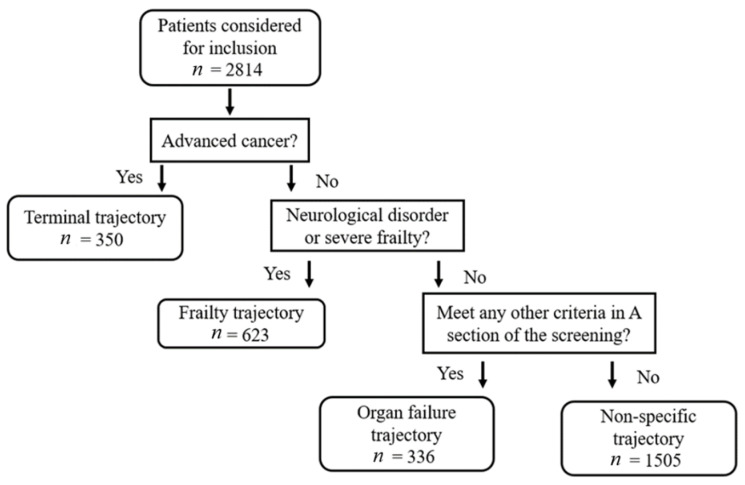
Flowchart of study participants and hierarchical model for trajectory classification.

Patients were categorized into three end-of-life trajectories (terminal cancer, organ failure, and frailty) according to their main illness using a screening tool established beforehand [20]. In the present study, we used the criteria defined by the screening tool. The terminal trajectory cohort was defined as patients with advanced cancer, metastatic, or locally aggressive disease. The frailty trajectory was defined as patients with advanced central neurological disease or very severe frailty. Advanced central neurological disease was defined by accompanying conditions of being long-term bed-bounded combined with repeated or severely progressive deterioration or recurrent pneumonia, shortness of breath, or respiratory failure requiring hospital admission; very severe frailty was defined by clinical traits of complete dependence, near end-of-life, and with clinical frailty scale score CSHA-CSF > scale 8 and 9. Patients diagnosed with any other illness on the screening chart besides terminal or frailty related illness were classified as the organ failure cohort. Patients that did not meet any illness description on the screening chart were classified as “other” and excluded from the study. A hierarchical model was employed in case patients had more than one possible trajectory [16]. The terminal trajectory supplanted the frailty trajectory, which supplanted the organ failure trajectory. The study flowchart is shown in Figure 1.

#### 2.2.2. Palliative Care Eligibility

Eligibility was assessed with the screening tool developed by George et al. [21] and further modified by Yang et al. [20]. The screening tool contained 15 items, 9 of which assessed the presence of an acute severe life-limiting illness, and 6 of which assessed unmet palliative care needs. It took approximately 15 min to complete the assessment for inclusion or exclusion of study patients. If a patient had one or more acute severe life-limiting illnesses accompanied with two or more items of unmet palliative care needs, they were considered eligible for palliative care. For patients eligible for palliative care, board-certified intensivists accredited by the Joint Committee of Intensive Care Medicine in Taiwan discussed options regarding palliative care and do-not-resuscitate (DNR) orders with the patient, their family members, or both.

#### 2.2.3. Timing of Referral

Timing of referral was calculated as the interval between an individual’s date of death and date of palliative care referral in the EICU. Timing of referral for patients with prior palliative referrals was defined as the timing of assessment at the EICU as well. Based on previous literature proposing similar timeframes, we chose 90 days before death as the cutoff point of early palliative care referral [2,22]; within 30 days before death as the cutoff point of late referral [23]; and 7 days or less before death as the cutoff point of very late referral [5,24].

#### 2.2.4. Charlson Comorbidity Index (CCI)

The CCI included 19 conditions as a weighted index to predict risk of death within one year of hospitalization for patients with specific comorbid conditions. Higher scores indicated a greater risk of death [25].

#### 2.2.5. Acute Physiology and Chronic Health Evaluation II (APACHE II)

APACHE II was used to measure the severity of disease for adults admitted to the intensive care unit. It measured 12 routine physiologic measurements (range 0 to 71). Higher scores indicated a greater severity of disease [26].

#### 2.2.6. Other Variables

Data on the patient’s age, sex, educational level, marital status, living arrangement, religion, frequency of ED visits or hospitalization within the past 6 months, DNR order status, length of stay, and hospital expenses were obtained from medical records.

### 2.3. Statistical Analysis

Data were analyzed using SPSS version 24.0 (IBM Corp., Armonk, NY, USA). Categorical variables were assessed with Pearson’s chi-squared tests among different trajectory cohorts, and continuous variables were assessed with a one-way analysis of variance. Multivariable logistic regression analyses were carried out to predict the acceptance of palliative care and mortality of patients upon ED admission. Prognostic indicators of palliative care acceptance included sociodemographic traits, end-of-life trajectory, CCI, APACHE II score, and unexpected hospital visits within the past 6 months. Prognostic indicators of inpatient mortality included age, sex, end-of-life trajectory, CCI, APACHE II score, and unexpected hospital visits in the past 6 months. Survival was defined as the duration from the day of palliative screening at the EICU to the day of death (in days). The Kaplan–Meier estimate was used to measure the probabilities of patients in the three trajectory cohorts living for a certain amount of time after palliative care screening/referral.

## 3. Results

In total, 2814 patients were admitted to the EICU during the study period. Of all the patients, 350 were included in the terminal cancer cohort (12.4%), 623 were included in the frailty cohort (22.1%), 336 were included in the organ failure cohort (11.9%), and 1505 were classified as “other” (53.5%). Patients not included in the terminal cancer, frailty, or organ failure cohorts were excluded from the study.

### 3.1. Sociodemographic Traits among Trajectories

Table 1 presents the data on the patients’ sociodemographic characteristics by end-of-life trajectory. Terminal patients had a lower mean age (71.4 ± 14.6) compared with patients with frailty and organ failure (87.6 ± 10.0 and 83.9 ± 12.6, respectively). Male patients were the majority in all three trajectories (terminal: 58.9%; frailty: 66.5%; organ failure: 66.1%). Most patients lived with family (89.7% of terminal patients, 73.5% of frailty patients, and 77.6% of organ failure patients). Patients with frailty (32.5%) and patients with organ failure (31.0%) were more likely to be widowed compared with terminal patients (15.4%). Terminal patients had higher levels of education than the patients with frailty and patients with organ failure. For all three trajectories, the majority of patients reported having no religion (36.9% of terminal; 41.3% of frailty; and 38.8% of organ failure).

### 3.2. Clinical Traits among Trajectories

Table 2 presents the data on the patients’ clinical characteristics. Based on the assessment criteria, more than 90% of patients from all three trajectories were qualified for palliative care. A higher percentage of eligible patients from the terminal trajectory (61.4%) were accepted for palliative care. Among the three trajectories, patients with frailty (54.6%) had the highest percentage of signed DNR orders before admission, whereas patients with organ failure (14.9%) had a relatively high percentage of patients without signed DNR orders. Across the three end-of-life trajectories, 90-day mortality was highest among terminal patients (63.4%), followed by patients with frailty (42.7%) and patients with organ failure (39.0%). Late referral was more prevalent among terminal patients (47.4%) compared with patients with frailty (34.0%) and patients with organ failure (30.1%). Very late referral was more prevalent among terminal patients (27.1%) compared with patients with frailty (21.2%) and patients with organ failure (18.8%). In terms of prognostic indicators, terminal patients showed more serious cases of comorbidity (CCI score: 8.17 ± 2.66) but less acute distress (APACHE II score: 18.66 ± 8.57), whereas patients with frailty showed less comorbidity (CCI score: 6.59 ± 2.05) but more severe acute physiological signs (APACHE II score: 24.44 ± 7.43). In the previous six months, more than half of the terminal patients had more than three ER visits or hospitalizations due to unexpected causes, which was significantly higher than the other two cohorts (*p* < 0.001). Across the three trajectories, terminal patients had significantly higher ER-related expenditures and average inpatient daily expenses (*p* < 0.001 and *p* < 0.001, respectively).

### 3.3. Referral Acceptance Indicator

The results of the binary logistic regression revealed that the only and significant predictor for palliative care referral acceptance was end-of-life trajectory (Table 3). Terminal patients had 1.7 odds of accepting a palliative care referral when taking other covariates into account.

### 3.4. Mortality Indicator

Table 4 presents the results of the binary logistic regression for 90-day mortality. The significant predictors for 90-day mortality included decreased age (adjusted odds ratio (aOR) 0.982, 95% confidence interval (CI) 0.972–0.993, *p* = 0.001), terminal trajectory (terminal vs. organ failure aOR 2.482, 95% CI 1.730–3.561, *p* < 0.001), increased CCI score (aOR 1.12, 95% CI 1.063–1.179, *p* < 0.001), and increased APACHE II score (aOR 1.045, 95% CI 1.029–1.062, *p* < 0.001), when taking other covariates into account.

### 3.5. Survival Pattern among Trajectories

**Figure 2:**
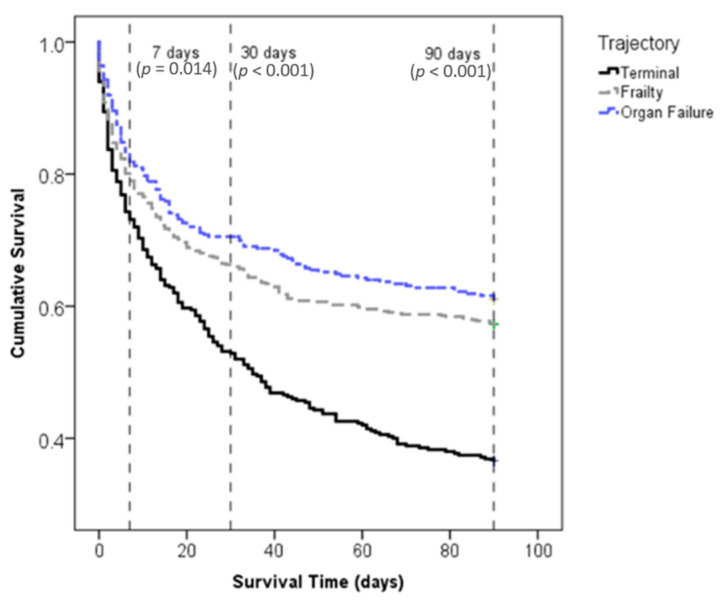
Kaplan–Meier survival curve for the three end-of-life trajectories.

A survival follow-up conducted 90 days after palliative assessment indicated that the percentage of survival decreased most rapidly in terminal patients, and that rapid death occurred among all trajectories within the first 30 days. Significant differences in probability of survival within 90 days were observed across the three end-of-life trajectories at time points of 7 (*p* = 0.014), 30 (*p* < 0.001), and 90 (*p* < 0.001) days (Figure 2).

## 4. Discussion

In this study, we observed the rate and timing of palliative care in 1309 patients with advanced and end-stage chronic conditions who sought emergent medical care at a 3000-bed medical center between 2018 and 2020. During the study period, the percentage of acceptance of palliative care referral in the EICU ranged from 50.1% among patients with frailty to 61.4% among terminal patients. Furthermore, approximately 20% of very late referrals and over 30% of late referrals for palliative care were observed among all trajectories, indicating the need for a more efficient referral response.

### 4.1. Palliative Care Acceptance

Previous studies have suggested that palliative referrals are determined by the medical resources available and patient preferences [27,28,29]. They have also suggested that patients’ preferences are more influenced by their sociodemographic characteristics and understanding of the prognosis than by their actual health status [29,30]. In contrast to prior studies, our findings suggested that patients’ sociodemographic characteristics and actual health status have limited influence on palliative care referral decisions. This finding may be related to the extensive national health coverage in Taiwan [31]. Equal coverage under the National Health Insurance Plan aids in overcoming social obstacles to healthcare, and mitigates the impact of sociodemographic inequality on healthcare access. Hence, in Taiwan, medical resource availability might not be the primary concern for patients’ needing life-extending treatment or palliative care. Similar to previous studies [3,32], the present study suggests that acceptance of palliative care referrals differs greatly by end-of-life trajectory. Differences in trajectory of functional decline and its predictability may explain our findings. It is worth noting that patterns of functional decline were different among end-of-life trajectories [15]. Unlike terminal cancer patients with sharp deteriorations and a clear terminal phase, patients with frailty or organ failure, diseases characterized by gradual decline, are less likely to acknowledge that their illness is terminal [15]. Insufficient information also increases patients’ decision conflicts [33]. As palliative care referral is associated with awareness and knowledge of the need for palliative care among the physician, patient, and family members [22,34], higher acceptance of palliative care referrals among cancer patients in our findings may reflect the differences in the degree of understanding of the terminal condition across different end-of-life trajectories. Patients with more knowledge of palliative care, with a more positive attitude toward palliative care, who view quality of life as the goal of end-of-life care, or have opted for a hospital death are more willing to receive palliative care at the end of life [35]. One cohort study that investigated cancer patients’ end-of-life preferences at multiple sites indicated that patients who had ever had an end-of-life discussion with their healthcare provider or who understood their illness was terminal were more likely to accept palliative services [29]. Clinicians should discuss palliative care early with the patient and family to allow them time to think about and discuss end-of-life care. This would avoid forcing the patient and family to make a quick decision at a critical moment. On the other hand, more palliative care campaigns that focus on the different end-of-life trajectories should be developed to raise public awareness of their needs and rights regarding palliative care. Palliative care referral in the ED is a new and developing area in Taiwan. Currently, ED physicians’ primary tasks are to quickly screen the needs for palliative care, ask patients or family members’ their palliative referral preference, and then make care plans. Not too much time was spared on discussion about goal of care with patients or their family members. Developing the Goal of Care (GOC) discussion guideline in the ED and professional training are further warranted.

### 4.2. Timing of Palliative Care

In the present study, over 20% of end-of-life patients had a very short referral time before death. Palliative care may be initiated as early as 180 days before a patient’s death [10,36]. Early referral to palliative care helps to clarify treatment preferences and goals of care, improves quality of life and symptom control, reduces distress, allows less aggressive care, and lowers spending [4,6,7,8,9,19,37], yet late referral remains common and palliative care is underutilized [5,8,9,34,38]. Patients with late referral to palliative care are less likely to benefit from multidisciplinary palliative care, and have more risk of a less dignified death [23,39,40]. Evidence shows that lower sociodemographic status, lack of a healthcare proxy, end-of-life trajectory marked by rapid and acute exacerbations, poor communication about the end of life, lack of prognostic tools for terminal illness, and undue optimism about the prospect of survival may contribute to late referral for palliative care [5,18,41,42]. Given that death is still a taboo subject in Chinese culture [43], some clinicians may object to explaining the progressive prognosis to the patient, which may result in the patient missing the opportunity of receiving palliative care near death. Additionally, absence of a formal prognostication and certification of near-death may cause ED clinicians to question the hopes and goals of the therapies that they initiate for the patient [44]. Accordingly, use of valid prognostic indicators related to end-of-life trajectories can help clinicians identify patients with advanced chronic conditions for a progressive palliative care approach [18,19,45]. In clinical settings, the APACHE II and CCI have been used to assess the patient’s overall and predicted condition. In the present study, age, end-of-life trajectory, CCI, and APACHE II were significant predictors for 90-day mortality (Table 4). Previous studies suggest that functional status is also a strong predictor for mortality rate [46,47]. Functional status has been integrated as a part of the screening criteria of the palliative care tool we used in this study [20], which allowed us to limit all patients’ functional status to a certain degree. However, future studies may consider functional status as another independent predictor when assessing palliative care needs. More to the point, survival patterns varied among trajectories and displayed different urgency for palliative care. Considering that each end-of-life trajectory differs, future research should consider these variables when measuring severity and progression to evaluate patients’ end-of-life trajectories. In this context, particular attention should be paid to the approximately 30% of patients who rejected palliative care referral and died within seven days or less after ED admission. Patients take a wide variety of factors into consideration, including physical, psychosocial, cultural, and family aspects, when making palliative care decisions [48]. However, palliative care referral in the present study mainly focused on patients’ physical severity and progression. Therefore, there is a need for future studies to explore the considerations of this specific group.

In the present study, more than 90% of patients who suffered from advanced, chronic, life-limiting illnesses were identified and referred for palliative care after admission in the ED, and nearly 20% of patient deaths occurred within seven days thereafter. These findings underscored the importance of early and appropriate palliative care referral so that the full benefits of palliative care are received by patients and families. In Taiwan, in order to provide people with a more comprehensive access to palliative care, we have launched different programs throughout different stages of medical consults. On the one hand, advance care planning policy, which was launched in 2019, allows individuals who are still in good health or with unidentified palliative care needs to discuss goals of care at an early stage. On the other hand, establishing ED palliative care guidelines and training of ED clinicians allow the ED to exert broader influence throughout the process of medical consults. The role of the ED has become prominently important, as it functions as both an early referral point and a last opportunity for palliative care. In the past, care in the ED often was focused on intensive, life-sustaining interventions, and the palliative care needs of patients with serious illness could go unmet [49,50]. Nowadays, as palliative care is recognized as a legitimate purpose of the ED, ED clinicians are more likely to initiate palliative care referrals, discuss palliative care concerns, and provide information and clarification [51]. As George et al. suggested [14], substantial work is yet to be done in terms of identifying ED patients in need of palliative care, training emergency medicine clinicians to provide high-quality primary palliative care, creating pathways to ED referrals for palliative care, and researching the outcomes and impact of palliative care provision on patients with serious illness in the ED. A previous study indicated that des pite surprise questions facilitating advance care planning discussions in the ED setting, the reliability of responses was a concern to physicians [52]. In this study, we adapted a more comprehensive screening tool that enables ED clinicians to extensively screen patients for unmet palliative care needs. Furthermore, because palliative care needs are complex and diverse, in addition to implementing a checklist approach, combination with educational initiatives and other system-change works may help identify palliative care needs of patients with different illness trajectories [53].

### 4.3. Study Limitations

This study had two major limitations. First, despite being a retrospective longitudinal study, data were drawn from a single medical center. Therefore, the results cannot be generalized beyond that particular setting. Selection bias may account for the negative correlation between the prediction of 90-day mortality rate and age. Using nationwide data in future studies would help to establish the full picture of palliative care referral in the ED in Taiwan. Second, given that most patients admitted to the ED meant to seek aggressive end-of-life care and still had potentially unmet palliative care needs, we included all qualified patients regardless of prior palliative care referral. We could not rule out the possibility of including individuals with prior palliative care referral in this study. Thus, the present study revealed the high demand for palliative care referral in the ED, and cannot explain the low palliative care provision and enrollment in general. The results, therefore, should be interpreted with caution.

## 5. Conclusions

In conclusion, the current study demonstrated that presenting trajectories as a framework can shed light on the improvements needed in timing and appropriateness of palliative care referral in the ED. In the time-pressured ED environment, acknowledgment of palliative-related characteristics of each trajectory can aid in quickly assessing the needs of palliative care for patients and initiating palliative care referrals. ED patients and their families would also benefit from appropriate palliative care referral and high-quality palliative care at the end of life.

## Figures and Tables

**Table 1:** Comparison of different end-of-life trajectories by patient sociodemographic characteristics.

Variable	Terminal *n* = 350 (%)	Frailty *n* = 623 (%)	Organ Failure *n* = 336 (%)	*p*
Age (y) *	71.4 ± 14.6	87.6 ± 10.0	83.9 ± 12.6	<0.001
Sex *				0.04
Female	144 (41.1)	209 (33.5)	114 (33.9)	
Male	206 (58.9)	414 (66.5)	222 (66.1)	
Living arrangement *				<0.001
With family	314 (89.7)	458 (73.5)	260 (77.4)	
At a healthcare facility	18 (5.1)	127 (20.4)	41 (12.2)	
Others	18 (5.1)	38 (6.1)	35 (10.4)	
Marital status *				<0.001
Married	243 (69.4)	369 (59.2)	189 (56.3)	
Single	53 (15.1)	51 (8.2)	43 (12.8)	
Widowed	54 (15.4)	203 (32.6)	104 (31.0)	
Educational status *				0.002
Below high school	171 (48.9)	364 (58.4)	194 (57.7)	
High school and above	177 (50.6)	242 (38.8)	135 (40.2)	
Others	2 (0.6)	17 (2.7)	7 (2.1)	
Religion *				0.004
Taoism	83 (23.7)	98 (15.7)	46 (13.7)	
Buddhism	117 (33.4)	198 (31.8)	122 (36.3)	
Catholic/Christian	17 (4.9)	63 (10.1)	33 (9.8)	
Others	4 (1.1)	6 (1.0)	4 (1.2)	
None	129 (36.9)	258 (41.4)	131 (39.0)	

Results expressed as number (%) for categorical variables and mean ± standard deviation for numerical variables; * *p* < 0.05 was considered statistically significant based on a Mann–Whitney U test or chi-squared test.

**Table 2:** Comparison of different end-of-life trajectories by patient clinical and HPC-related characteristics.

Variable	Terminal *n* = 350 (%)	Frailty *n* = 623 (%)	Organ Failure *n* = 336 (%)	*p*
HPC eligibility *	342 (97.7)	615 (98.7)	309 (92.0)	<0.001
Acceptance of HPC *				0.003
Accepted	210 (61.4)	308 (50.1)	165 (53.4)	
Rejected	132 (38.6)	307 (49.9)	144 (46.6)	
DNR order *				<0.001
Signed before assessment	153 (43.7)	340 (54.6)	139 (41.4)	
Signed after assessment	168 (48.0)	223 (35.8)	147 (43.8)	
Rejected	29 (8.3)	60 (9.6)	50 (14.9)	
Death within				
1 week *	95 (27.1)	132 (21.2)	63 (18.8)	0.022
1 month *	166 (47.4)	212 (34.0)	101 (30.1)	<0.001
3 months *	222 (63.4)	266 (42.7)	131 (39.0)	<0.001
>3 months *	128 (36.6)	357 (57.3)	205 (61.0)	<0.001
CCI score *	8.2 ± 2.7	6.6 ± 2.1	6.8 ± 2.4	<0.001
APACHE II *	18.7 ± 8.6	24.4 ± 7.4	22.7 ± 8.1	<0.001
Unexpected hospital visits *^,#^	195 (55.7)	239 (38.4)	100 (29.8)	<0.001
ED length of stay (h)	9.7 ± 16.9	9.8 ± 17.2	8.2 ± 9.5	0.26
EICU length of stay (h)	50.8 ± 31.2	52.3 ± 37.0	53.1 ± 35.1	0.69
Inpatient length of stay (d) ^∆^	21.1 ± 26.4	21.6 ± 24.1	21.9 ± 21.4	0.90
ED expense (NTD) *	23,440.1 ± 21,077.6	19,052.4 ± 8646.8	19,056.2 ± 9007.5	<0.001
Average inpatient expense (NTD/d) *	16,584.9 ± 13,964.3	13,592.1 ± 7159.2	14,890.8 ± 12,247.8	<0.001

Results expressed as number (%) for categorical variables and mean ± standard deviation for numerical variables. HPC = hospice and palliative care; DNR = do-not-resuscitate; CCI = Charlson Comorbidity Index; APACHE = Acute Physiology and Chronic Health Evaluation; ED = emergency department; EICU = emergency intensive care unit; NTD = New Taiwan dollar; * *p* < 0.05 was considered statistically significant based on a Mann–Whitney U test or chi-squared test; ^#^ visited the ED or hospitalized ≥3 times in the previous six months due to unpredicted causes; ^∆^ length of stay from single hospitalization after ED admission to death or discharge.

**Table 3:** Binary logistic regression analyses for acceptance of hospice and palliative care.

Variable	aOR	95% CI	*p*
Age (by 1-year increment)	0.997	(0.985–1.008)	0.57
Sex (male to female)	0.984	(0.750–1.291)	0.91
End-of-life trajectory			
Terminal *	1.712	(1.203–2.438)	0.003
Frailty	0.993	(0.755–1.306)	0.96
Organ Failure	1.0	reference	
CCI (by 1-point increment)	0.990	(0.942–1.041)	0.70
APACHE II (by 1-point increment)	1.012	(0.997–1.027)	0.13
Unexpected hospital visits	1.017	(0.808–1.279)	0.88
Living arrangement			
With family	0.931	(0.585–1.482)	0.77
Health facilities	0.819	(0.483–1.390)	0.46
Others	1.0	reference	
Marital status			
Married	0.871	(0.586–1.297)	0.50
Widowed	0.811	(0.515–1.278)	0.37
Single	1.0	reference	
Education status			
Below high school	0.881	(0.383–2.027)	0.77
High school and above	0.809	(0.348–1.882)	0.62
Others	1.0	reference	
Religion			
Taoism	0.854	(0.612–1.192)	0.35
Buddhism	1.004	(0.770–1.309)	0.98
Catholic/Christian	1.063	(0.698–1.618)	0.78
Muslim	2.281	(0.204–25.541)	0.50
Others	3.995	(0.842–18.965)	0.08
None	1.0	reference	

CCI = Charlson Comorbidity Index; APACHE = Acute Physiology and Chronic Health Evaluation; aOR = adjusted odds ratio; CI = confidence interval; * *p* < 0.05 was considered statistically significance in the regression model.

**Table 4:** Binary logistic regression analysis of clinical predictors for 90-day inpatient mortality.

Variable	aOR	95% CI	*p*
Age (by 1-year increment) *	0.982	(0.972–0.993)	0.001
Sex (male to female)	0.982	(0.773–1.246)	0.88
End-of-life trajectory			
Terminal *	2.482	(1.730–3.561)	<0.001
Frailty	1.156	(0.874–1.529)	0.31
Organ Failure	1.0	reference	
CCI (by 1-point increment) *	1.12	(1.063–1.179)	<0.001
APACHE II (by 1-point increment) *	1.045	(1.029–1.062)	<0.001
Unexpected hospital visits ^#^	1.111	(0.878–1.406)	0.38

CCI = Charlson Comorbidity Index; APACHE = Acute Physiology and Chronic Health Evaluation; aOR = adjusted odds ratio; 95% CI = confidence interval; * *p* < 0.05 was considered statistically significance in regression model; ^#^ visited the emergency department or hospitalized ≥3 times in the previous six months due to unpredicted causes.

## Data Availability

The data presented in this study are available on request from the corresponding author.
